# Distinct DNA methylation epigenotypes in bladder cancer from different Chinese sub-populations and its implication in cancer detection using voided urine

**DOI:** 10.1186/1755-8794-4-45

**Published:** 2011-05-20

**Authors:** Pi-Che Chen, Ming-Hsuan Tsai, Sidney KH Yip, Yeong-Chin Jou, Chi-Fai Ng, Yanning Chen, Xiaoling Wang, Wei Huang, Chun-Liang Tung, Gary CW Chen, Martin MS Huang, Joanna HM Tong, Eing-Ju Song, De-Ching Chang, Cheng-Da Hsu, Ka-Fai To, Cheng-Huang Shen, Michael WY Chan

**Affiliations:** 1 Department of Urology, Chia-Yi Christian Hospital, Chia-Yi, Taiwan; 2 Department of Life Science, National Chung Cheng University, Min-Hsiung, Chia-Yi, Taiwan; 3 Institute of Molecular Biology, National Chung Cheng University, Min-Hsiung, Chia-Yi, Taiwan; 4 Department of Surgery, The Chinese University of Hong Kong, Hong Kong, China; 5 Department of Pathology, Institute of the Forth Hospital of Hebei Medical University, Shijiazhuang, China; 6 Department of Medicine, Huaqiao Hospital, Jinan University, Guang zhou, China; 7 Department of Pathology, Chia-Yi Christian Hospital, Chia-Yi, Taiwan; 8 Department of Anatomical and Cellular Pathology, State Key Laboratory in Oncology in South China, The Chinese University of Hong Kong, Hong Kong, China; 9 Department of Bioscience Technology, Chang Jung Christian University, Tainan, Taiwan; 10 Department of Medical Research, Chia-Yi Christian Hospital, Chia-Yi, Taiwan

## Abstract

**Background:**

Bladder cancer is the sixth most common cancer in the world and the incidence is particularly high in southwestern Taiwan. Previous studies have identified several tumor-related genes that are hypermethylated in bladder cancer; however the DNA methylation profile of bladder cancer in Taiwan is not fully understood.

**Methods:**

In this study, we compared the DNA methylation profile of multiple tumor suppressor genes (*APC*, *DAPK*, *E-cadherin*, *hMLH1*, *IRF8*, *p14*, *p15*, *RASSF1A*, *SFRP1 *and *SOCS-1*) in bladder cancer patients from different Chinese sub-populations including Taiwan (104 cases), Hong Kong (82 cases) and China (24 cases) by MSP. Two normal human urothelium were also included as control. To investigate the diagnostic potential of using DNA methylation in non-invasive detection of bladder cancer, degree of methylation of *DAPK*, *IRF8*, *p14*, *RASSF1A *and *SFRP1 *was also accessed by quantitative MSP in urine samples from thirty bladder cancer patients and nineteen non-cancer controls.

**Results:**

There were distinct DNA methylation epigenotypes among the different sub-populations. Further, samples from Taiwan and China demonstrated a bimodal distribution suggesting that CpG island methylator phentotype (CIMP) is presented in bladder cancer. Moreover, the number of methylated genes in samples from Taiwan and Hong Kong were significantly correlated with histological grade (P < 0.01) and pathological stage (P < 0.01). Regarding the samples from Taiwan, methylation of *SFRP1*, *IRF8*, *APC *and *RASSF1A *were significantly associated with increased tumor grade, stage. Methylation of *RASSF1A *was associated with tumor recurrence. Patients with methylation of *APC *or *RASSF1A *were also significantly associated with shorter recurrence-free survival. For methylation detection in voided urine samples of cancer patients, the sensitivity and specificity of using any of the methylated genes (*IRF8*, *p14 *or *sFRP1*) by qMSP was 86.7% and 94.7%.

**Conclusions:**

Our results indicate that there are distinct methylation epigenotypes among different Chinese sub-populations. These profiles demonstrate gradual increases with cancer progression. Finally, detection of gene methylation in voided urine with these distinct DNA methylation markers is more sensitive than urine cytology.

## Background

Bladder cancer is the sixth most common cancer in the world [1] and tenth most common cancer in Taiwan. Majority of bladder cancer is comprised of urothelial carcinoma (UC) (also known as transitional cell carcinoma, TCC). The incidence of urothelial cancer is particularly high in southwestern coast of Taiwan [2] thus suggesting that UC in these areas may have unique carcinogenesis pathway. Although the carcinogenesis process is unclear so far, accumulation of multiple genetic and epigenetic alternations leading to the activation of proto-oncogenes and/or inactivation of tumor-suppressor genes (TSGs) is a common consensus [3-5].

One of the distinctive features of UC is that over 80% of newly diagnosed cases are non-invasive superficial lesions; however, 50% of them will recur accompanied with advanced stage of disease and poor prognosis. UC patients will then need to have a long-term follow-up with repeated urine cytology and invasive cystoscopy for recurrence monitoring. Conventional urine cytology has been the standard noninvasive method for cancer detection and disease monitoring; however, the sensitivity of this method is known to be low, especially for low-grade UC. Therefore, a more sensitive, non-invasive method for cancer detection is required.

Epigenetic alteration such as DNA methylation is an important mechanism in regulating transcription [6]. Gene promoter methylation plays an important role in normal mammalian development; yet, aberrant promoter hypermethylation is frequently observed in human cancers and displays a non-random tumor specific DNA methylation pattern [7,8]. It is thus suggested that this non-random methylation pattern may be related to the unique signaling pathways that are dysregulated in response to specific carcinogens exposed in specific tumor type [9]. Therefore, DNA methylation may be a promising method for non-invasive cancer detection [10].

We along with others have previously shown that methylation of multiple tumor suppressor genes can be observed in bladder cancer patients as well as its corresponding voided urine samples [11-15]. In order to develop a sensitive epigenetic markers panel for diagnosis and prognosis in this locality, a more comprehensive epigenetic profile of UC in Taiwan is needed.

In this study, we aimed to compare the DNA methylation profile of mutilple tumor suppressors in bladder cancer paitents from Taiwan, Hong Kong and China by methylation specific PCR (MSP). The diagnostic potential of the genes that were found to be frequently methylated in samples from Taiwan were further accessed by quantitative MSP (qMSP). Our result demonstrated that there were distinct methylation epigenotypes in bladder urothelial cancer patients from different Chinese sub-populations and that methylation markers may provide a sensitive strategy for non-invasive cancer detection in urine samples.

## Methods

### Tissue and urine samples

Tissue samples from a total of 104 bladder UC patients from Chia-Yi Christian Hospital, Taiwan, 82 samples from Prince of Wales Hospital, Hong Kong and 24 samples from Forth Hospital of Hebei Medical University, China were collected. For urine samples, paired voided urine from thirty bladder cancer patients were collected retrospectively. In addition, nineteen urine samples from age- and sex-matched non-cancer controls were also included. All urine samples were collected from Chia-Yi Christian Hospital, Taiwan. Urine samples were processed for DNA extraction as described previously [11]. All patients were asked to sign the informed consent for obtaining the specimens. The clinical pathological data for the tissue and urine samples are summarized in table 1 and 2 respectively. Tumors were graded and staged according to the WHO grading [16] and the AJCC TNM staging system [17] respectively. As a control, primary culture of normal urothelium from two individuals (purchased from ScienCell, Carlsbad, CA) were used in this study. All studies involving human samples were conformed to the Helsinki Declaration and approved by the Institutional Review Boards of the Chia-Yi Christian Hospital and the Clinical Research Ethics Committee of the Chinese University of Hong Kong and Hebei Medical University.

**Table 1:** Summary of clinical-pathological data of tumor samples

	Taiwan (n = 104)	Hong Kong (n = 82)	China (n = 24)
**Age**			
Median	70.5	73	64.5
Range	40 - 92	46 - 92	43 - 80
**Gender**			
Male	84	60	23
Female	20	22	1
**Histological Grade**			
Grade 1	34	16	8
Grade 2	42	41	12
Grade 3	28	25	4
**Pathological Stage**			
Stage Ta	41	38	8
Stage T1	41	21	6
Stage ≥ T2	22	23	10
**Relapse**			
Primary	66	23	ND
Recurrence	38	58	ND

ND: data not available

**Table 2:** Summary of clinical-pathological data of urine samples from tumor patients and normal control in Taiwan

	Cancer (n = 30)	Normal (n = 19)
**Age**		
Median	71.5	62
Range	47 - 92	39 - 85
**Gender**		
Male	25	11
Female	5	8
**Histological Grade**		
Grade 1	12	
Grade 2	13	
Grade 3	5	
Pathological Stage*		
Stage Ta	13	
Stage T1	12	
Stage ≥ T2	4	
**Relapse**		
Primary	26	
Recurrence	4	

*Staging information of one cancer patient is not available

### Extraction of DNA from paraffin-embedded tissues and urine samples

DNA was extracted from formalin-fixed, paraffin-embedded tissues or from voided urine sediment using Tissue and Cell Genomic DNA Purification Kit (Genemark, Taipei, Taiwan) according to manufacturer protocol. H&E-stained sections from each tumor sample were examined by an experienced pathologist to confirm the clinicopathological parameters.

### Bisulfite modification and Methylation-Specific PCR (MSP)

DNA was bisulfite-modified using EZ DNA Methylation Kit (ZYMO research, Orange, CA) as described previously [18]. For MSP reaction, four μl of bisulfite-converted DNA were amplified in a total volume of 20 μl containing Platinum Taq DNA polymerase (Invitrogen, Carlsbad, CA). Primers for the MSP reaction are shown in table S1 (Additional file 1). CpGenome Universal Methylated DNA (IVD) (Millipore, Bedford, MA) was used as positive control for methylation, while human peripheral lymphocyte DNA amplified by GenomePlex Complete Whole Genome Amplification Kit (Sigma-Aldrich, St Louis, MO) was used as a positive control for unmethylation and water was used as negative control for PCR. Ten μl of PCR products were loaded onto 10% non-denaturing polyacrylamide gel. The gel was then stained with ethidium bromide, and visualized under UV illumination. Methylated samples are defined as the presence of methylated PCR products in that samples.

### Real-time quantitative methylation-specific PCR (qMSP) in urine samples

For detection of gene promoter methylation in urine samples, a more sensitive and quantitative real-time methylation-specific PCR (qMSP) was used as described [18]. In detail, qMSP reactions were performed using ABI Stepone real time PCR system (ABI, Foster city, CA) in a total volume of 20 μl containing 10 μl of 2X SYBR Green Real-time PCR Master Mix (Toyobo, Osaka, Japan), 160 nM of each primers and 4 μl of bisulphite converted DNA at 95°C for 10 mins, 40 cycles of 95°C for 15 sec, 60°C for 30 sec, and 72°C for 30 sec. Primers for qMSP targeting *DAPK*, *IRF8*, *p14*, *RASSF1A *and *SFRP1 *are shown in table S1 (additional file 1). A region of *β-actin*, devoid of any CpG dinucleotide was used to normalize for input DNA using the following primer pairs: ACTB-forward 5' TGGTGATGGAGGAGGTTTAGTAAGT and ACTB-reverse, 5' AACCAATAAAACCT ACTCCTCCCTTAA. The amount of methylated DNA was determined by the threshold cycle number (Ct) for each sample against a standard curve generated by SSSI-treated DNA-MSP cloned fragment. The percentage of methylation of a certain gene was calculated as the ratio of amount of methylated gene/ACTB in a sample divided by the same ratio of SssI-treated sperm DNA and multiplied by 100 [18].

### Statistical analysis

Comparison of non-parametric variables was assessed by Kruskal-Wallis Test or Mann-Whitney test whichever appropriate. Association between clinical-pathological parameters was assessed by χ^2 ^or Fisher's exact test. Methylation index, MI is defined as the number of methylation gene divided by the total number of gene studied in a sample as previously described [19]. MI≥ 5 is considered as high. Cut-off value for qMSP in urine samples was determined by ROC curved (Additional file 2: Figure S1). Recurrence-free survival (RFS) was calculated from the date of surgery to the date of recurrence or last follow-up date and assessed by Kaplan-Meier analysis using log-rank test. All statistical analysis was performed by SPSS version 13.0 for windows (SPSS, Chicago, IL, USA). P < 0.05 was considered as significant.

## Results

### Methylation profile of tumor suppressors in bladder UC

**Figure 1:**
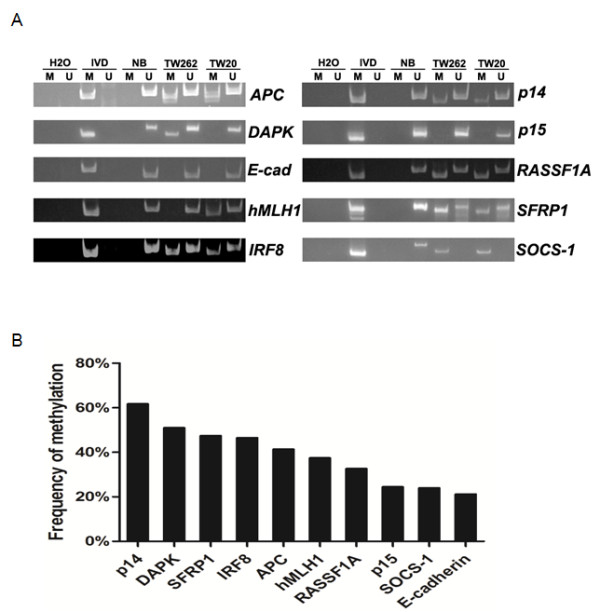
**Methylation analysis of *APC, DAPK, E-cadherin, hMLH1, IRF8, p14, p15, RASSF1A, SOCS-1 *and *SFRP1 *in tumor tissues of bladder cancer patients by MSP**. A. Representative gel electrophoresis images of the MSP result from Taiwan bladder cancer samples TW262 and TW20. M indicates the presence of methylated genes; U indicates the presence of unmethylated genes. IVD (*in vitro *methylated DNA) was used as the positive control for methylation; Normal blood (NB) genomic DNA was used as the positive control for unmethylation; B. Frequency of methylation of different genes in tumor tissues from 104 Taiwan bladder cancer patients.

**Figure 2:**
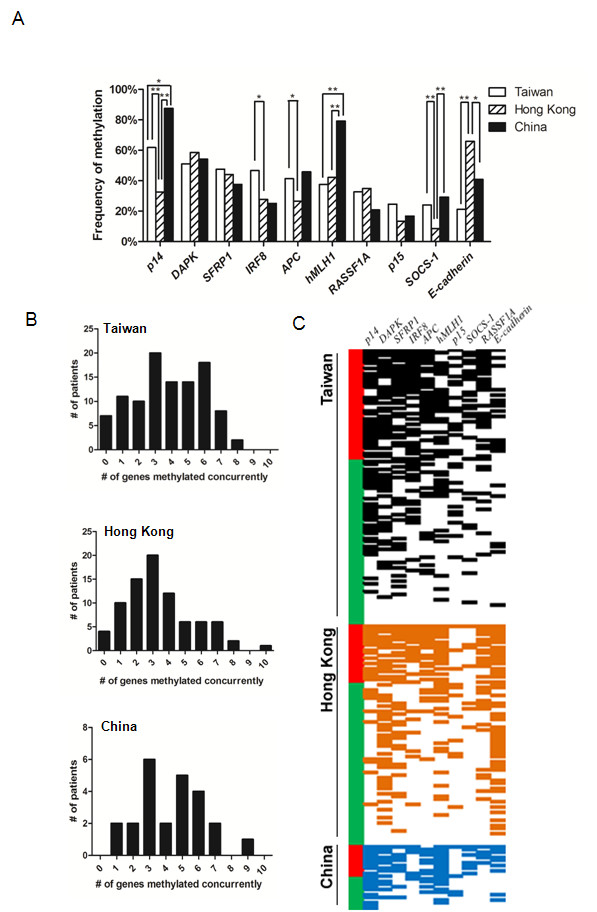
**DNA Methylation profiles from different Chinese sub-populations**. A. The histogram showed that methylation of *p14, IRF8, APC, hMLH1, SOCS-1 *and *E-cadherin *varied among different Chinese sub-populations. * and ** denote P < 0.05 and P < 0.01 respectively (Mann-Whitney U). B, Histogram showing the number of patients against the number of genes methylated concurrently. Samples from Taiwan and China displayed bimodal distribution which is a characteristic of CpG island methylator phentotype (CIMP). C. Dichotomous heat map of the DNA methylation data from different Chinese sub-populations. Black, orange and blue blocks represented methylated loci from Taiwan, Hong Kong and China respectively. Red bars indicated MI-high populations (≥5 genes showing concurrent methylation) and green bars indicated MI-low populations.

We have analyzed the methylation frequency of multiple tumor suppressors (*APC*, *DAPK*, *E-cadherin*, *hMLH1*, *IRF8*, *p14*, *p15*, *RASSF1A*, *SFRP1 *and *SOCS-1*) that are found to be frequently methylated in various human cancer including bladder cancer [11,20-25] in primary bladder UC tissues from Taiwan (104 cases), Hong Kong (82 cases) and China (Beijing, 24 cases) by methylation-specific PCR (Figure 1A). Samples from Taiwan showed that frequent methylation was detected in *p14 *(61.8%), *DAPK *(51.0%), *SFRP1 *(47.5%), and *IRF8 *(46.6%), while methylation was also detected in *APC *(41.4%), *hMLH1 *(37.5%), *RASSF1A *(32.7%), *p15 *(24.5%), *SOCS-1 *(24.0%), and *E-cadherin *(21.2%) (Figure 1B). Regarding samples from Hong Kong, frequent methylation of *E-cadherin *(65.9%), *DAPK *(58.5%), *SFRP1 *(44.0%), and *hMLH1 *(42.2%) were detected. Moreover, frequent methylation of *p14 *(87.5%), *hMLH1 *(79.2%), *DAPK *(54.2%), *APC *(45.8%), and *E-cadherin *(40.9%) were detected in samples from China (Figure 2A).

Notably, none of these genes showed any aberrant hypermethylation in normal control as demonstrated in this study (Additional file 3: Figure S2) and previously [11] thus suggesting it is tumor specific event.

### Distinct DNA methylation epigenotypes in bladder cancer patients from different Chinese sub-populations

Compared with different Chinese sub-populations, there were different methylation frequency among samples from Taiwan, Hong Kong and China (Figure 2). Significant differences between methylation of *APC*, *E-cadherin*, *hMLH1*, *IRF8*, *p14 *and *SOCS-1 *were found (Figure 2A). Interestingly, samples from Taiwan displayed a bimodal distribution in terms of number of methylated genes which is the characteristic of CpG island methylation phenotype, CIMP [26-28] (Figure 2B). However, such phenomenon was not observed in samples from Hong Kong. Although samples from China also displayed such bimodal distribution, the potential biased from the relatively small sample size from this locality cannot be excluded. Additionally, in light of the fraction of methylated genes or methylation index (MI) in a sample, two methylation groups could be observed: MI-high and MI-low groups (Figure 2C). MI-high groups displayed a similar pattern regardless of sample locality, yet MI-low group exhibited a locality-specific methylation pattern. Taken together, these data suggest that CIMP may exist in bladder cancer samples at least in samples from Taiwan and that there are distinct DNA methylation epigenotypes among samples from Taiwan, Hong Kong and China.

### Gene methylation gradual increases with cancer progression

**Figure 3:**
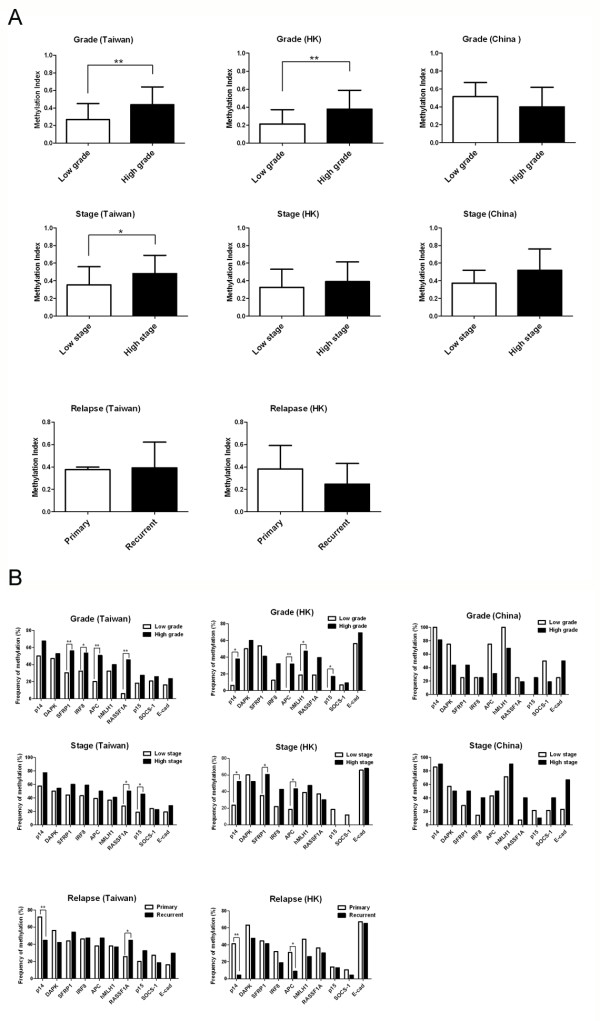
**Association between gene methylation and tumor progression of bladder cancer in different Chinese sub-populations**. The histogram demonstrated the association between (A) methylation index, MI or (B) methylation of individual gene with tumor progression in terms of tumor grade, stage and relapse in bladder cancer samples from Taiwan, Hong Kong (HK) and China. Low grade represented grade 1 cases; high grade represented ≥ grade 2 cases. Low stage represented stage < T2 cases; high stage represented ≥ T2 cases. * and **, P < 0.05 and P < 0.01 respectively.

To investigate the relationship between DNA methylation and tumor progression, we analyzed the methylation index (MI) of the samples with reference to clinical-pathological parameters. Regarding samples from Taiwan, high histological grade and pathological stage was significantly associated with higher MI (grade, P < 0.01; stage, P < 0.05; Figure 3A). However, tumor recurrence was not associated with MI. Samples with high MI were also significantly associated with higher grade and stage (P < 0.001) (Table 3). Similar tendencies could also be observed in samples from Hong Kong (grade, P < 0.05; Figure 3A, Additional file 4: Table S2). However, such correlation was not observed in samples from China where sample size was relatively small (Figure 3, Additional file 4: Table S2). Besides, we have also analyzed the association between methylation of individual genes and tumor progression. Methylation of *SFRP1*, *IRF8*, *APC *and *RASSF1A *were significantly associated with increased tumor grade and stage in samples from Taiwan (Figure 3B). Methylation of *RASSF1A *was also associated with tumor recurrence (Figure 3B and Table 4). Besides, significant association between increased tumor grade, stage or tumor recurrence and methylation of *p14*, *SFRP1*, *APC*, *hMLH1 *and *p15 *were observed in samples from Hong Kong. Surprisingly, methylation of *p14 *and *APC *were inversely correlated with tumor recurrence (Figure 3B). In summary, consistent with previous findings, our results suggest that DNA methylation increases gradually with tumor progression [29,30].

**Table 3:** Correlations between methylation index and clinical-pathological data in Taiwan samples

	**MI - High**^**1**^	MI - Low	
	Mean ± SD		
		
**Age**	70.4 ± 12.7	68.4 ± 11.8	
	No. of cases		*P*
	
**Gender**			
Male	36	48	0.295
Female	6	14	
**Histological Grade**			
Grade 1	5	29	
Grade 2	16	26	<0.001
Grade 3	21	7	
**Pathological Stage**			
Stage Ta	9	32	
Stage T1	17	24	<0.001
Stage ≥ T2	16	6	
**Relapse**			
Primary	24	42	0.273
Recurrence	18	20	

^1 ^Cases grouped into MI-High if methylation index ≥ 5; otherwise, grouped into MI-Low

**Table 4:** Correlation between *RASSF1A *methylation and cancer recurrence in Taiwan samples

	*RASSF1A*		
		
	Methylated	unmethylated	P
Primary (n = 66)	17 (25.7%)^1^	49 (74.2%)	
Recurrence (n = 38)	17 (44.7%)	21 (55.2%)	0.039

^1^Values are number of cases (%)

### Methylation of APC and RASSF1A predict recurrence free survival in bladder cancer patients

**Figure 4:**
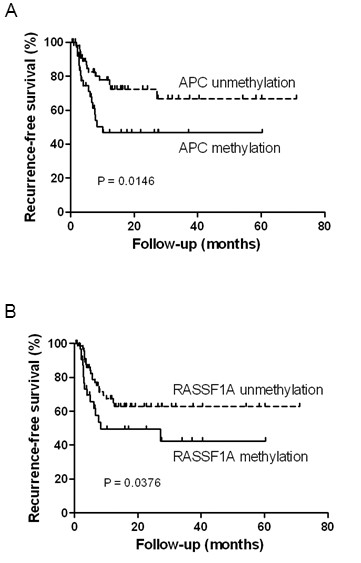
**Kaplan-Meier analysis of *APC *or *RASSF1A *methylation for recurrence-free survival (RFS) in bladder cancer patients from Taiwan**. Patients were grouped according to methylation of (A) APC or (B) RASSF1A as determined by MSP. Patients with methylation of APC or RASSF1A have a significant shorter time of RFS. P-values from Gehan-Breslow-Wilcoxon test are shown.

Recurrence is a common clinical manifestation in bladder UC, thus development of a relapse indicator will be important for cancer monitoring. We investigated the correlation between methylation of the analyzed markers and recurrence-free survival (RFS) of bladder cancer patients by Kaplan-Meier analysis. Since MI did not show any correlation with RFS in our samples, we proceeded to analyze such correlation with individual methylation markers. Out of the 10 methylation makers, patients in Taiwan with methylation of *APC *(P = 0.0146) or *RASSF1A *(P = 0.0376) demonstrated a shorter RFS than those without methylation (Figure 4).

### DNA methylation markers in voided urine as cancer detection tool

**Figure 5:**
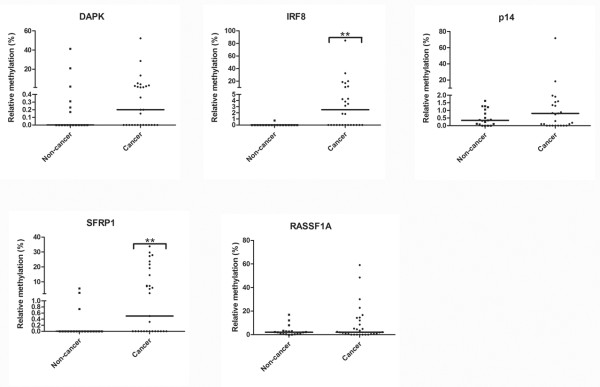
**Quantification of *DAPK, IRF8, p14, RASSF1A *and *SFRP1 *methylation in voided urine samples from bladder cancer patients from Taiwan by qMSP**. Values in dot plot represented methylation percentage relative to the IVD (100%). The horizontal line represented median value. ** denotes P < 0.01 (Mann-Whitney U).

In order to assess the feasibility of using DNA methylation as a biomarker for cancer detection and recurrence monitoring, genes showing highest methylation frequency (*p14*, *DAPK*, *SFRP1*, and *IRF8*) in tumor tissues from Taiwan samples and *RASSF1A *which have been previously found to be methylated in bladder cancer patients [12,22,31] were selected as potential targets for methylation detection in urine samples that were acquired retrospectively from a different patient pool from Taiwan. Voided urine from thirty bladder UC patients were analyzed by a more sensitive quantitative MSP (qMSP) assay for methylation detection [32,33]. Nineteen samples from non-cancer patients in the same locality were also included as control (Table 2). As compare to non-cancer group, higher methylation level of *DAPK*, *IRF8*, *p14*, *RASSF1A *and *SFRP1 *were detected in cancer samples (Figure 5). We then performed receiver operating characteristic (ROC) curve to determine an optimal cut-off values for each gene (Additional File 2: Figure S1). Based on these cut-off values, the sensitivity of our qMSP assay using *DAPK*, *IRF8*, *p14*, *RASSF1A *and *SFRP1 *was 26.7%, 56.7%, 27.6%, 30.0% and 41.4% respectively; and the specificity was 89.5%, 94.7%, 100%, 89.5% and 100% respectively (Table 5). By combining *IRF8*, *p14 *and *SFRP1 *together as a panel of methylation markers, the sensitivity and specificity of a sample showing methylation of one of these 3 genes was 86.7% and 94.7%, respectively (Table 5). Notably, the sensitivity of this markers panel for grade 1 and recurrent tumors was 91.7% and 100% respectively. Additionally, we have also analyzed the correlation between RFS and methylation of individual markers (or markers panel) in these thirty urine samples. However, probably due to small sample size, no significant difference can be found. A more detail methylation analysis on urine samples can also be found in table S3 (Additional file 5).

**Table 5:** Sensitivity and specificity of cancer detection using voided urine samples from Taiwan

	*DAPK*	*IRF8*	*p14*	*RASSF1A*	*SFRP1*	**Methylation markers**^**1**^
**Sensitivity (%)**						
**All cases**(n = 30)	26.7	56.7	27.6	30.0	41.4	86.7
**Grade 1**(n = 12)	25.0	50.0	41.7	16.7	50.0	91.7
**Grade 2-3**(n = 18)	27.7	61.1	16.7	38.9	33.4	83.3
**Primary **(n = 26)	30.8	61.5	30.8	30.8	34.6	84.6
**Recurrence **(n = 4)	0	25	0	25	75	100
**Specificity (%)**(n = 19)	89.5	94.7	100	89.5	100	94.7

^1 ^Any one of these genes (*IRF8*, *p14 *and *sFRP1*) showed methylation in urine samples.

## Discussion

Aberrant promoter methylation is a hallmark of cancer. In this study, we analyzed the methylation profiles of ten tumor suppressors that are frequently methylated in various human cancers [11,20-25] in bladder UC from different Chinese sub-populations. Our results showed that 93.3%, 95.2% and 100% of patients from Taiwan, Hong Kong and China, respectively, had at least one gene methylated thus suggesting that epigenetic event of gene methylation is frequent in bladder cancer. Although none of these genes showed any aberrant hypermethylation in primary culture of normal urothelium from two individuals, more control samples may be needed for further validation. On the other hand, methylation of the analyzed genes in patients from Hong Kong and/or China showed an obvious diversity as compared to that from Taiwan; for example, *IRF8 *showed higher frequency of methylation in samples from Taiwan (46.6%) than from Hong Kong (27.6%) and China (25.0%). However, the result from China patients may need to further validate by increasing the sample size. Nevertheless, these differences may be attributed to genetic or environmental differences in these localities as differences in sex, age, stage and grade cannot explain this methylation diversity.

Exposure to environmental carcinogen and uptake of different diets has been shown to be the major reasons causing such distinct DNA methylation epigenotypes [34,35]. In Taiwan, bladder cancer is particularly common in endemic areas of arsenic-induced Blackfoot disease. Previous studies have demonstrated that arsenic pollution is associated with *DAPK *and *RASSF1A *methylation in bladder cancer [36,37]. It may be one of the factors that contribute to this distinct methylation epigenotype. However, our result demonstrated a similar methylation level of *DAPK *and *RASSF1A *in samples from different Chinese sub-populations. It may result from the fact that arsenic exposure from artesian well water has decreased in arsenious-endemic area due to the improvement of drinking water system [38]. However, similar effect from different carcinogens in different localities cannot be excluded. Besides, it has also been reported that influences of dietary factors interact with DNA methylation in colorectal cancer [39]. Thus, different environmental factors together with genetic factors may contribute to these distinct methylation profiles.

In the current study, samples from Taiwan and China displayed a strongly bimodal distribution by the number of methylated genes, which implied that CIMP may exist in bladder UC [36]. However samples from Hong Kong did not exhibit such typical CIMP methylation pattern. This may be due to the fact that genes that we analyzed are not suitable for CIMP analysis in samples from Hong Kong, thus suggesting that the carcinogenesis in bladder UC in Hong Kong may be different from Taiwan and China fundamentally.

In keeping with previous observation [19,29], our study also demonstrated that methylation of several genes such as *APC *and *RASSF1A *were associated with tumor progression. Methylation of *RASSF1A *was also associated with tumor recurrence. However, the inverse correlation between methylation of *p14 *and *APC *with tumor recurrence needs to be further validated.

DNA methylation has been previously demonstrated to be able to predict patient's survival and recurrence [19,29,40]. In the current study, patients from Taiwan with methylation in *APC *or *RASSF1A *tended to have a shorter RFS. The absence of such correlations in samples from other Chinese sub-populations may be due to similar reasons as discussed above. Previous studies have indicated that bladder cancer patients with *APC *or *RASSF1A *methylation show a trend toward poor survival [19,29]. Furthermore, loss of E-cadherin expression had been reported to be associated with increased risk of recurrence in bladder cancer [41]. Although Muramaki *et al *did not investigate the role of DNA methylation in their study, loss of E-cadherin expression may result from aberrant promoter methylation. Our results also demonstrated a similar trend that the primary bladder UC patients with *E-cadherin *methylation had a shorter time of RFS (median = 6.1 months versus 10.9 months, P = 0.07).

Due to the high recurrence rate of bladder UC, patients usually need to have repeated cystoscopy for disease monitoring thus promoting the development of non-invasive strategies. With the advances of cancer epigenetics, detection of methylated genes in voided urine becomes feasible as previously demonstrated [11,14,15,23,42]. In this study, we utilized a more sensitive quantitative real-time MSP assay (qMSP) for cancer detection in voided urine samples using a combination of methylated markers. Methylation can be detected in low grade samples with high sensitivity. Importantly, methylation can be detected in all of the urine samples from patients with recurrent tumors. However, more samples and specific methylation markers should be included for further validation and improve the diagnostic accuracy. By combining urine cytology with methylation markers and other protein biomarkers such as NMP22 [43], the sensitivity of cancer detection can also be dramatically increased.

## Conclusions

Our study demonstrated that there were distinct DNA methylation epigenotypes in bladder cancer samples from different Chinese sub-populations. Detection of methylated genes in voided urine, as a potential non-invasive diagnostic tool, deserves further investigation.

## Competing interests

The authors declare that they have no competing interests.

## Authors' contributions

PCC, SSKY, YCJ, CFN, YC, XW, WH and CHS collected samples and performed experiments. MHT, GCC, MMH, JHT, performed experiments. MHT, KFT and MWYC performed statistical analysis. YC, XW, CLT and KFT provided pathological data. EJS, DCC and CDH participated in the design of the study. KFT and MWYC formulated and directed the study design. All authors read and approved the final manuscript.

## Pre-publication history

The pre-publication history for this paper can be accessed here:

http://www.biomedcentral.com/1755-8794/4/45/prepub

## Supplementary Material

Additional file 1 Table S1: Primer sequences, annealing temperatures and product size for MSP Click here for file

Additional file 2 **Figure S1: ROC curve of *DAPK, IRF8, p14, RASSF1A*, and *SFRP1 *methylation**. Receiver-operator characteristic (ROC) curve of the *DAPK, IRF8, p14, RASSF1A*, and *SFRP1 *methylation based on qMSP result. The Cut-off value and the corresponding sensitivity and specificity for each gene is also shown. Click here for file

Additional file 3 **Figure S2: MSP gel image of the studied tumor suppressors in normal human normal urothelium (HUC) from two individuals**. Methylation analysis of *APC, DAPK, E-cadherin, hMLH1, IRF8, p14, p15, RASSF1A, SOCS-1 *and *SFRP1 *in normal human normal urothelium (HUC) from two individuals. M indicates the presence of methylated genes; U indicates the presence of unmethylated genes. IVD (*in vitro *methylated DNA) was used as the positive control for methylation and water (H_2_O) was used as a negative control for PCR. Click here for file

Additional file 4 Table S2: Correlations between methylation index and clinical-pathological parameters in bladder cancer samples of different Chinese sub-population Click here for file

Additional file 5 Table S3: Summary of qMSP analysis of voided urine samples from Taiwan Click here for file
