# Supplementary material for: Distinct DNA methylation epigenotypes in bladder cancer from different Chinese sub-populations and its implication in cancer detection using voided urine
Source: BMC Med Genomics. 2011 May 20;4:45. doi: 10.1186/1755-8794-4-45 (PMC3127971; doi:10.1186/1755-8794-4-45)
Supplement: Additional file 1 — Table S1: Primer sequences, annealing temperatures and product size for MSP [file 1755-8794-4-45-S1.DOC]

**Table S**1 Primer sequences, annealing temperatures and product size for MSP

| Gene | Primer sequences (5’-3’) | Annealing temperature  (°C) | Product  Size  (bp) |
| --- | --- | --- | --- |
| *APC* | M F: TATTGCGGAGTGCGGGTC  R: TCGACGAACTCCCGACGA  U F: GTGTTTTATTGTGGAGTGTGGGTT  R: CCAATCAACAAACTCCCAACAA | 60  60 | 98  108 |
| *DAPK* | M F: GGATAGTCGGATCGAGTTAACGTC  R: CCCTCCCAAACGCCG  U F: GGAGGATAGTTGGATTGAGTTAATGTT  R: CAAATCCCTCCCAAACACCAA | 60  60 | 98  106 |
| *E-cadherin* | M F: TTAGGTTAGAGGGTTATCGCGT  R: TAACTAAAAATTCACCTACCGAC  U F: TAATTTTAGGTTAGAGGGTTATTGT  R: CACAACCAATCAACAACACA | 64  58 | 116  97 |
| *hMLH1* | M F: ACGTAGACG TTT TATTAGGGTCGC  R: CCTCATCGTAACTACCCGCG  U F: TTTTGATGTAGATGTTTTATTAGGGTTGT  R: ACCACCTCATCATAACTACCCACA | 60  60 | 115  124 |
| *IRF8* | M F: attttcggggttgttcgttc  R: cacctaaaatccaaaaacgacg  U F: gtatttttggggttgtttgttt  R: ctcacacctaaaatccaaaaacaaca | 60  62.5 | 120  126 |
| *p14* | M F: GTGTTAAAGGGCGGCGTAGC  R: AAAACCCTCACTCGCGACGA  U F: TTTTTGGTGTTAAAGGGTGGTGTAGT  R: CACAAA AACCCTCACTCACAACAA | 66  66 | 122  132 |
| *p15* | M F: GCGTTCGTATTTTGCGGTT:  R: CGTACAATAACCGAACGACCGA  U F: TGTGATGTGTTTGTATTTTGTGGTT  R: CCATACAATAACCAAACAACCAA | 62  62 | 148  154 |

Table S1 (continued)

| *RASSF1A* | M F: GTGTTAACGCGTTGCGTATC  R: AACCCCGCGAACTAAAAACGA  U F: TTTGGTTGGAGTGTGTTAATGTG  R: CAAACCCCACAAACTAAAAACAA | 60  60 | 94  108 |
| --- | --- | --- | --- |
| *SOCS-1* | M F: TTCGCGTGTATTTTTAGGTCGGTC  R: CGACACAACTCCTACAACGACCG  U F: TTATGAGTATTTGTGTGTATTTTTAGGTTGGTT  R: CACTAACAACACAACTCCTACAACAACCA | 60  60 | 160  175 |
| *SFRP1* | M F: TGTAGTTTTCGGAGTTAGTGTCGCGC  R: CCTACGATCGAAAACGACGCGAACG  U F: GTTTTGTAGTTTTTGGAGTTAGTGTTGTGT  R: CTCAACCTACAATCAAAAACAACACAAACA | 64  64 | 126  135 |
