# Supplementary figures and images for: Distinct DNA methylation epigenotypes in bladder cancer from different Chinese sub-populations and its implication in cancer detection using voided urine
Source: BMC Med Genomics. 2011 May 20;4:45. doi: 10.1186/1755-8794-4-45 (PMC3127971; doi:10.1186/1755-8794-4-45)

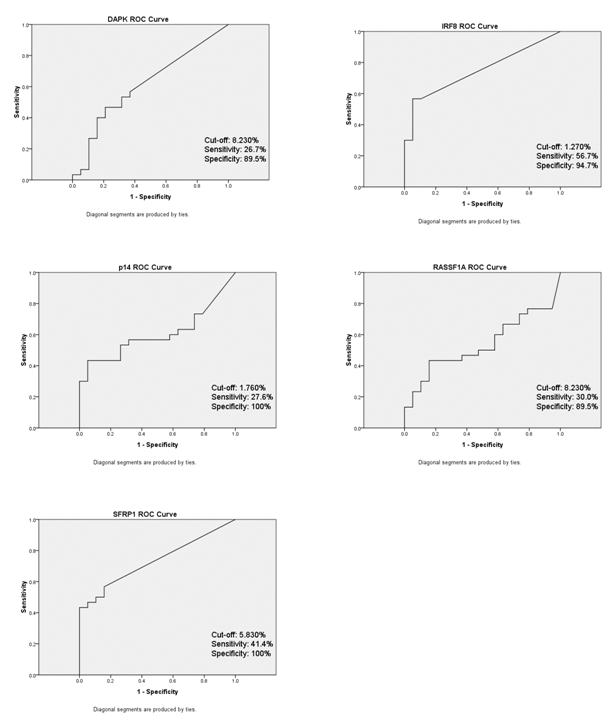

Supplement: Additional file 2 — Figure S1: ROC curve of DAPK, IRF8, p14, RASSF1A, and SFRP1 methylation. Receiver-operator characteristic (ROC) curve of the DAPK, IRF8, p14, RASSF1A, and SFRP1 methylation based on qMSP result. The Cut-off value and the corresponding sensitivity and specificity for each gene is also shown. [file 1755-8794-4-45-S2.TIFF]

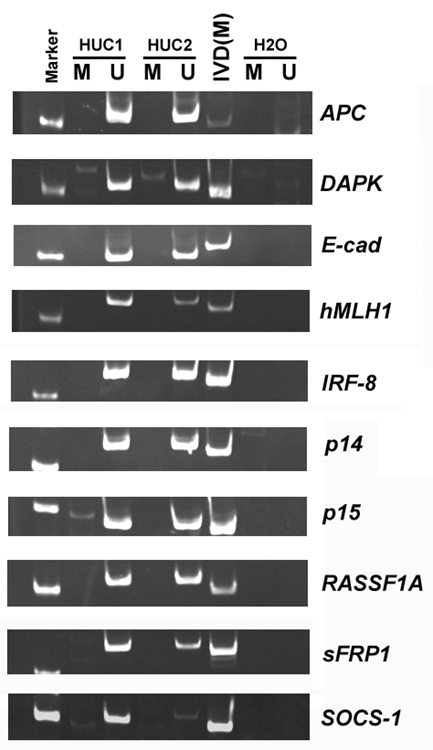

Supplement: Additional file 3 — Figure S2: MSP gel image of the studied tumor suppressors in normal human normal urothelium (HUC) from two individuals. Methylation analysis of APC, DAPK, E-cadherin, hMLH1, IRF8, p14, p15, RASSF1A, SOCS-1 and SFRP1 in normal human normal urothelium (HUC) from two individuals. M indicates the presence of methylated genes; U indicates the presence of unmethylated genes. IVD (in vitro methylated DNA) was used as the positive control for methylation and water (H2O) was used as a negative control for PCR. [file 1755-8794-4-45-S3.TIFF]
