# Supplementary material for: Distinct DNA methylation epigenotypes in bladder cancer from different Chinese sub-populations and its implication in cancer detection using voided urine
Source: BMC Med Genomics. 2011 May 20;4:45. doi: 10.1186/1755-8794-4-45 (PMC3127971; doi:10.1186/1755-8794-4-45)
Supplement: Additional file 4 — Table S2: Correlations between methylation index and clinical-pathological parameters in bladder cancer samples of different Chinese sub-population [file 1755-8794-4-45-S4.DOC]

**Table S2** Correlations between methylation index and clinical-pathological parameters in bladder cancer samples of different Chinese sub-population

|  | Taiwan (n=104) | | |  | Hong Kong (n=82) | | |  | China (n=24) | | |
| --- | --- | --- | --- | --- | --- | --- | --- | --- | --- | --- | --- |
|  | MI-High^1^ | MI-Low | P-value^4^ |  | MI-High | MI-Low | P-value |  | MI-High | MI-Low | P-value |
| **Age** | 70.4 ± 12.7^2^ | 68.4 ± 11.8 | 0.547 |  | 72.0 ± 9.5 | 71.3 ± 10.1 | 0.692 |  | 62.5 ± 11.0 | 64.8 ± 11.4 | 0.814 |
| **Gender** |  |  |  |  |  |  |  |  |  |  |  |
| Male | 36^3^ | 48 | 0.295 |  | 16 | 44 | 0.478 |  | 11 | 12 | 0.5 |
| Female | 6 | 14 |  |  | 5 | 17 |  |  | 1 | 0 |  |
| **Histological Grade** |  |  |  |  |  |  |  |  |  |  |  |
| Grade 1 | 5 | 29 | <0.001 |  | 1 | 15 | 0.04 |  | 5 | 3 | 0.333 |
| Grade 2-3 | 37 | 33 |  |  | 20 | 46 |  |  | 7 | 9 |  |
| **Pathological Stage** |  |  |  |  |  |  |  |  |  |  |  |
| Stage Ta | 9 | 32 | <0.001 |  | 8 | 30 | 0.481 |  | 2 | 6 | 0.165 |
| Stage T1 | 17 | 24 |  |  | 5 | 16 |  |  | 3 | 3 |  |
| Stage ≧T2 | 16 | 6 |  |  | 8 | 15 |  |  | 7 | 3 |  |
| **Relapse** |  |  |  |  |  |  |  |  |  |  |  |
| Primary | 24 | 42 | 0.273 |  | 18 | 40 | 0.079 |  |  |  |  |
| Recurrence | 18 | 20 |  |  | 3 | 20 |  |  |  |  |  |

^1^ Cases grouped into MI-High if methylation index ≧5; otherwise, grouped into MI-Low

^2^ mean ± SD

^3^ number of case

^4^ Chi-square or Fisher exact test was used wherever appropriate except for age where Mann-Whitney U-test was used.
